# Young persimmon fruit extract as a multifunctional natural additive in ice cream: Effects on physical properties, antioxidant functionality, and sensory acceptability

**DOI:** 10.1016/j.fochx.2026.104662

**Published:** 2026-10-23

**Authors:** Miyuna Kobayashi, Tomoyoshi Hara, Akari Sunahara, Mika Ishigaki, Yoko Tsurunaga

**Affiliations:** a Faculty of Human Sciences, Shimane University, Matsue 690-8504, Shimane, Japan; b Institute of Agricultural and Life Sciences, Academic Assembly, Shimane University, Matsue 690-8504, Shimane, Japan; c Graduate School of Human and Social Sciences, Shimane University, Matsue 690-8504, Shimane, Japan; d The United Graduate School of Agricultural Sciences, Tottori University, 4-101 Koyama-Minami, Tottori 680-8553, Japan

**Keywords:** Condensed tannin, Ice cream, Tannin–protein interaction, Melting resistance, Microstructure, Sensory evaluation, Young persimmon fruit

## Abstract

Young persimmon fruit extract (YPE), a tannin-rich fruit-thinning by-product, was added to ice cream at added soluble tannin content (STC) levels of 0.0–1.0%. YPE imparted a natural reddish-brown color and significantly enhanced antioxidant activity without substantially altering pH or Brix. Microstructural, particle size, viscosity, and overrun analyses indicated that tannin–milk protein interactions densified the matrix and reduced air incorporation, improving melting resistance: complete melting required 51–54 min at 0.1–0.5% (peaking at 0.3%) added STC and 75 min at 1.0% added STC, versus 42 min for the control. Hardness, adhesiveness, and gumminess increased with added STC. Overall acceptability was maintained up to 0.5% added STC but declined at 1.0%, indicating a practical range of 0.1–0.5%. Exploratory sandwich ELISA showed a modest, concentration-dependent reduction in detectable casein and β-lactoglobulin content (up to 26%). YPE is a promising clean-label, multifunctional additive for valorizing an agricultural by-product.

## Highlights

• Persimmon extract from fruit thinning gave natural color and antioxidant activity.
• Tannin–milk protein interactions likely densified the matrix and reduced overrun.
• Melting time extended from 42 to 51–54 min at 0.1–0.5% (peaking at 0.3%) added STC without stabilizers.
• Overall acceptability was maintained up to 0.5% added STC but declined at 1.0%.
• Detectable milk allergen content decreased modestly with increasing added STC.

## 1 Introduction

Recent years have seen growing interest in plant-derived polyphenols not only for their health-promoting properties, including antioxidant and anti-inflammatory activities (Adal & Aktar, 2024; Amatori et al., 2016), but also as functional ingredients that modify food texture and stabilize food structures (Cosme et al., 2025; Kaur et al., 2025). Among polyphenols, tannins in particular have been widely reported to exert such texture-modifying and structure-stabilizing effects (Girard & Awika, 2020; Wicks et al., 2023). Tannins are a class of polyphenols broadly distributed throughout the plant kingdom and are classified into two structural groups based on their molecular architecture: hydrolysable tannins and condensed tannins. The present study focuses on condensed tannins, specifically proanthocyanidins. Condensed tannins possess multiple phenolic hydroxyl groups and form stable complexes with proteins through hydrogen bonding, hydrophobic forces, electrostatic interactions, and van der Waals forces (Feng et al., 2023).

Although tannin–protein interactions have traditionally been viewed negatively as causes of astringency and inhibition of protein digestibility (Horigome et al., 1988; McRae & Kennedy, 2011), recent food science research has actively explored their practical applications. These include technologies that combine tannin–protein interactions with polysaccharides to control astringency (Lou et al., 2019), and the potential use of complex formation between tannins and allergenic proteins to mask or shield antigenic epitopes (Unterhauser et al., 2023), thereby reducing immunogenicity (C. Zhang et al., 2024). Furthermore, tannins are abundant in agricultural by-products generated during processing and harvesting (Fraga-Corral et al., 2021), making them attractive from a circular economy perspective as a means of adding value to otherwise discarded materials.

Ice cream represents a complex multiphase colloidal system where milk proteins play a key role in stabilizing fat globules, ice crystals, and air cells (Goff, 2002). Critical quality attributes such as overrun, product density, and melting behavior are strongly governed by these microstructural components. While plant extracts and polyphenolic natural colorants are increasingly utilized to enhance functional attributes, their impact on ice cream physical quality is non-linear. Yingwong et al. (2026) demonstrated that copigmented anthocyanins enhance mix viscosity and air retention within an optimal concentration range (1–3%), whereas higher levels compromise matrix integrity and accelerate melting. Furthermore, multivariate analyses by Pangastuti et al. (2025) highlight how distinct extract formulations differentially influence fat globule size, overrun, and melting resistance. Despite these insights, a comprehensive understanding of how specific tannin–milk protein interactions induce physical changes in microstructural parameters such as overrun and density, and how these changes relate to ultimate sensory acceptability, remains insufficient.

While bovine milk proteins, broadly categorized into caseins (up to 80%) and whey proteins (up to 20%), offer high nutritional value, both fractions contain major allergens that trigger severe immunoglobulin E (IgE)-mediated reactions (Villa et al., 2018). The casein fraction (Bos d 8) comprises α_s1_-, α_s2_-, β-, and κ-caseins, which predominantly feature heat-stable linear IgE-binding epitopes. In contrast, the whey fraction includes major allergens such as β-lactoglobulin (β-LG) and α-lactalbumin (α-LA), which possess both well-characterized conformational and linear epitopes that collectively contribute to overall milk allergenicity. Conventional hypoallergenization approaches such as enzymatic hydrolysis and prolonged heat treatment are associated with limitations including bitter taste generation and residual antigenicity (Lara-Villoslada et al., 2005). While polyphenol-mediated reduction of allergen detection has been reported for soy protein (Qiu et al., 2025), whether similar effects occur for milk proteins in a complex, low-temperature, multiphase dairy matrix such as ice cream remains unclear.

The present study therefore aimed to comprehensively evaluate the effects of a tannin-rich extract from young (unripe) persimmon fruit, a currently underutilized agricultural by-product, on the physical properties, antioxidant functionality, and sensory acceptability of ice cream. Specifically, this study aimed to (i) identify, among several plant-derived tannin sources, one with high binding affinity for the major milk proteins; and (ii) characterize the resulting changes in ice cream color, microstructure, texture, melting resistance, and antioxidant activity, thereby assessing the potential of young persimmon fruit extract (YPE) as a functional, clean-label additive that adds value to an otherwise discarded fruit-thinning by-product. As a secondary, exploratory objective, this study also examined whether the resulting tannin–protein interactions could concurrently reduce milk allergen detectability, given that such masking effects have been reported for other polyphenol–protein systems.

## 2 Materials and methods

### 2.1 Experiment 1: Screening of plant materials based on tannin–protein interactions

#### 2.1.1 Plant materials and preparation of tannin extracts

The sixteen plant materials used in this study (representing twelve plant species) were identified through a preliminary in-house screening of approximately 300 plant species/parts, collected from the campus of Shimane University and the Shimane Agricultural Technology Center, in which tannin content was assessed using the Folin–Ciocalteu method. These sixteen materials exhibited the highest tannin content among those screened and were therefore used for further evaluation of their protein-binding capacity. The common names, scientific names, and plant parts used were as follows: Ginkgo (*Ginkgo biloba*: fruits), *Styrax obassia*, Red bayberry (*Morella rubra*: buds, young leaves), Japanese chestnut ‘Porotan’ (*Castanea crenata* ‘Porotan’: immature burrs), Grape ‘Shinku’ (*Vitis* ‘Shinku’: young fruits), Silk tree (*Albizia julibrissin*: leaves), Buddhist pine (*Podocarpus macrophyllus*: leaves), White oak (*Quercus myrsinifolia*: young leaves, mature leaves), Oak (*Quercus serrata*: hulls, fruits), Persimmon (*Diospyros kaki*: calyxes, young fruits), Chinese juniper (*Juniperus chinensis*: leaves), Japanese mallotus (*Mallotus japonicus*: leaves).

Each plant material was freeze-dried and pulverized into a fine powder using a mill (ABS-W, Osaka Chemical Co., Ltd., Osaka, Japan). To prepare the tannin extracts, 1.0 g of each powder was mixed with 19 mL of distilled water in an Erlenmeyer flask, heated in a boiling water bath for 10 min, immediately ice-cooled, and filtered.

#### 2.1.2 Preparation of milk protein solutions

Two milk proteins, whey protein and casein, were used, and solutions were prepared at a final protein concentration of 20 mg/mL. The whey protein solution was prepared by dissolving whey protein concentrate (WINZONE Whey Protein Perfect Choice, Plain Flavor, Nippon Shinyaku Co., Ltd., Kyoto, Japan; protein content approximately 72%) in distilled water. The casein solution was prepared by dissolving casein (Cat. No. 07319–82, Nacalai Tesque, Inc., Kyoto, Japan) in 1.5 M Tris buffer (pH 8.8).

#### 2.1.3 Mixing of tannin extracts with milk proteins and heat treatment

A 200 μL aliquot of each protein solution was dispensed into a 1.5 mL microtube and heated at 95 °C for 10 min. After cooling, 200 μL of the tannin extract prepared in Section 2.1.1 was added to each tube (distilled water was added to the control). The mixture was heated again at 95 °C for 10 min and then cooled on ice for 10 min. Insoluble aggregates were pelleted by centrifugation (6708 ×*g*, 1 min), and the resulting supernatant was used for SDS-PAGE analysis.

#### 2.1.4 SDS-PAGE analysis

SDS-PAGE analyses for both the screening experiment (Section 2.1) and the ice cream protein extracts (Section 2.2) were performed according to the method of Laemmli (1970) with slight modifications, using precast polyacrylamide gels (e-PAGEL 5–20%, *E*-T520L, ATTO Co., Tokyo, Japan) run at a constant voltage of 300 *V*, molecular weight markers (XL-Ladder Broad, AproScience Co., Ltd., Kyoto, Japan), and staining with One-Step CBB solution (CBB-1000, AproScience Co., Ltd.).

For the screening experiment, the supernatant obtained from the tannin–milk protein mixtures (Section 2.1.3) was diluted with distilled water to a protein concentration of 2 mg/mL, mixed in equal volume with Laemmli sample buffer (Bio-Rad Laboratories, Inc., Hercules, CA, USA) containing 5% 2-mercaptoethanol (final protein concentration, 1 mg/mL), and denatured at 95 °C for 5 min using an aluminum block thermostat (DTU-1CN, Taitec Corporation, Koshigaya, Japan). Electrophoresis was performed using a mini electrophoresis unit (WSE-1165, ATTO Co.) with a 5–20% precast gel; 5.0 μL of marker and 8.5 μL of sample were loaded per well. Gel images were acquired using a scanner (GT-X980, Seiko Epson Corporation, Suwa, Japan).

For the ice cream protein extracts, samples were prepared using the FASPEK ELISA II® extraction buffer (Morinaga Institute of Biological Science), 0.07 M phosphate buffer (pH 7.0) containing 0.1 M KCl, and simulated digestive fluids representing the oral, gastric, and intestinal phases according to the standardized INFOGEST in vitro digestion protocol (Brodkorb et al., 2019). Briefly, samples were sequentially incubated at 37 °C in the oral phase (simulated salivary fluid with α-amylase, 75 U/mL; 2 min), gastric phase (simulated gastric fluid with pepsin, 2000 U/mL; pH 3.0; 2 h), and intestinal phase (simulated intestinal fluid with pancreatin, 100 U/mL trypsin activity, and bile, 10 mmol/L; pH 7.0; 2 h). At the end of each phase, enzymatic reactions were stopped by cooling on ice followed by heating at 70 °C for 40 min. Protein concentration was adjusted to 1 mg/mL, mixed in equal volume with 2× Laemmli sample buffer containing 5% 2-mercaptoethanol (final protein concentration, 0.5 mg/mL), and heated at 95 °C for 5 min. Electrophoresis was performed using a 5–20% precast gel; 3.5 μL of marker and 6.0 μL of sample (corresponding to 4.25 μg protein per lane) were loaded per well.

### 2.2 Experiment 2: Production and evaluation of ice cream

#### 2.2.1 Materials and preparation of young persimmon fruit extract

Based on the screening results (Section 2.1), young persimmon (*Diospyros kaki*) fruit was selected as the additive material. Harvested young fruits were freeze-dried and pulverized, and the powder was suspended in distilled water and autoclaved at 120 °C for 20 min (LSX-300, Tomy Seiko Co., Ltd., Tokyo, Japan). After filtration, the extract was centrifuged at 1000 ×*g* for 3 min (LCX-100, Tomy Seiko Co., Ltd.), and the resulting supernatant, which had a soluble tannin content (STC) of 1050 mg catechin equivalent (CE)/100 mL (1.05%, *w*/*v*), was designated as YPE and stored at 4 °C until use.

Autoclaving was selected because it allowed simultaneous extraction of water-soluble components and sterilization of the extract in a single step, which was considered essential for food-safety purposes given the intended use in a dairy product. However, such severe thermal treatment (120 °C, 20 min) can hydrolyze condensed tannins and alter their degree of polymerization relative to the fresh fruit, even when the total STC (expressed as CEs) remains largely unchanged (Chamorro et al., 2012). Other heat-labile constituents of the fruit may also be altered by this treatment; therefore, YPE should be regarded as a heat-treated extract rather than as representative of the composition of the fresh young fruit.

#### 2.2.2 Ice cream manufacturing

Five treatment groups were established at STC addition levels of 0.0 (Control), 0.1, 0.3, 0.5, and 1.0% by mixing YPE (STC: 1050 mg CE/100 mL) and distilled water to a total liquid volume of 400 mL. A commercially available ice cream dry mix (Morinaga Dry Mix CP, Morinaga Milk Industry Co., Ltd., Tokyo, Japan) (200 g) was dissolved in the liquid phase under continuous stirring, heated to 95 °C, and immediately cooled to 2 °C or below in an ice bath (Ruger et al., 2002), yielding the ice cream base. According to the manufacturer's specifications, the dry mix contained 20.2% fat, 11.3% protein, 63.7% carbohydrates (including sugars), and 0.51% salt equivalent, and included emulsifier as additives. The cooled base was processed in an ice cream maker (IC*E*-PRO100J, Cuisinart, Stamford, CT, USA) for 60 min and immediately collected for evaluation.

#### 2.2.3 Sample preparation for measurements

Immediately after freezing, the ice cream was transferred to a pre-cooled bowl, packed into containers according to the measurement item, and stored at −18 °C (Akalin et al., 2018; Wang et al., 2026): stainless-steel dishes (filled without air gaps and leveled) for color, texture, and overrun; silicone ice cube molds for melting behavior; and lidded stainless-steel containers for antioxidant activity and sensory evaluation. The photograph was taken with a digital camera (E-M5 Mark III, OM Digital Solutions, Tokyo, Japan) using fixed settings (ISO 200, f/5.6, 1/800 s, fixed white balance) against a black background under 5500 K lighting.

#### 2.2.4 Determination of soluble tannin content and antioxidant activity

A 5 g portion of melted ice cream was mixed with 20 mL of 50% methanol and agitated at 40 °C for 2 h, and the extract was diluted as appropriate for each assay. Retained STC was determined using the Folin–Ciocalteu method as described by Kumagai et al. (2026) and expressed as mg CE/100 g of ice cream.

Antioxidant activity was evaluated using three complementary assays. The hydrophilic oxygen radical absorbance capacity (H-ORAC) was determined according to Watanabe et al. (2012) with minor modifications: the diluted extract was mixed with 75 mM phosphate buffer (pH 7.4), fluorescein, and 2,2′-azobis(2-amidinopropane) dihydrochloride (AAPH) in a 96-well plate, and fluorescence (excitation 485 nm, emission 520 nm) was monitored every 2 min for 90 min at 37 °C using a microplate reader (SH-9000Lab, Corona Electric Co., Ltd., Ibaraki, Japan). The 2,2-diphenyl-1-picrylhydrazyl (DPPH) radical scavenging activity was measured according to Tsurunaga et al. (2022): the diluted extract was mixed with a 2-(N-morpholino) ethanesulfonic acid (MES) buffer–DPPH solution, incubated in the dark at room temperature for 20 min, and the absorbance was measured at 540 nm. The 2,2′-azino-bis(3-ethylbenzothiazoline-6-sulfonic acid) (ABTS) radical scavenging activity was determined according to Aimone et al. (2023) with modifications: the ABTS radical solution (7 mM ABTS and 140 mM potassium peroxodisulfate, kept in the dark for 12–16 h and diluted with ethanol to an absorbance of 0.70 ± 0.02 at 734 nm) was mixed with the diluted extract, incubated at 30 °C for 4 min, and the absorbance was measured at 734 nm. For all three assays, results were expressed as μmol Trolox equivalent (TE)/g of ice cream.

#### 2.2.5 Color measurement

Color was measured following the method of Park et al. (2015) to avoid measurement error caused by lens condensation at low temperatures. Samples were thawed at room temperature (25 °C), filled into disposable cells, and measured using a colorimeter (Color Reader CR-13, Konica Minolta, Inc., Tokyo, Japan). Color was expressed as L* (lightness), a* (redness/greenness), b* (yellowness/blueness), and C* (chroma). Measurements were performed five times per sample (*n* = 5).

#### 2.2.6 Scanning electron microscopy (SEM)

The microstructure of the ice cream (including ice crystal distribution and air cell structure) was evaluated by SEM following freeze-drying of the samples. Although cryo-SEM is generally recommended for frozen food observation, conventional SEM of freeze-dried samples was employed due to instrumental constraints. This approach relies on the principle that the porous matrix formed by sublimation of ice during freeze-drying reflects the size and spatial distribution of the original ice crystals, and void morphology in freeze-dried samples has therefore been widely used as a proxy for ice crystal structure (Pérez-Bermúdez et al., 2023). Nevertheless, this proxy approach carries known limitations: freeze-drying artifacts such as matrix shrinkage and localized structural collapse can occur, particularly under slow freezing rates, and pore dimensions can be influenced by the cooling/drying conditions themselves rather than solely by the original ice crystal size (Pérez-Bermúdez et al., 2023; Voda et al., 2012). To minimize confounding from sample-preparation variability, all samples in this study (control and all STC addition levels) were freeze-dried under identical conditions, so that comparisons of relative differences in void morphology among treatment groups remain valid even if absolute pore dimensions may not perfectly correspond to the in situ ice crystal size.

Cross-sections of freeze-dried ice cream samples were affixed to SEM specimen stubs (Type-HM, Nissin EM Co., Ltd., Tokyo, Japan) using double-sided carbon tape (8 mm × 20 m). After gold sputter coating, observations were performed using a scanning electron microscope (Miniscope TM3000, Hitachi High-Tech Corporation, Tokyo, Japan) at an accelerating voltage of 20 kV and magnifications of ×250 and ×500.

#### 2.2.7 Particle size distribution

The particle size distribution of the samples was determined using a laser diffraction particle size analyzer (Partica LA-960 V2, Horiba, Ltd., Kyoto, Japan). Prior to measurement, approximately 10 g of melted ice cream was dissolved in 10 mL of 50% methanol and further diluted to a final dilution factor of 200. Each sample was vortexed immediately prior to introduction into the analyzer. Water was used as the dispersant with a refractive index of 1.35, and no ultrasonic treatment was applied. The median diameter (D_50_, μm) and volume-mean diameter (μm) were recorded as indices of particle size distribution. Measurements were performed three times per sample (*n* = 3).

#### 2.2.8 Overrun, density, and viscosity

Overrun was determined gravimetrically by weighing the ice cream base (prior to freezing) and the ice cream packed into a stainless-steel dish of fixed volume, and calculated using the following equation:

Overrun (%) = $\frac{W_{\mathrm{base}}-W_{\mathrm{ice}}}{W_{\mathrm{ice}}}\times 100$

where *W*_*base*_ and *W*_*ice*_ are the weights of the ice cream base and the ice cream, respectively, filling the same container (*n* = 5). Density was calculated by dividing the mass of the ice cream, measured immediately after removal from a − 18 °C freezer, by the volume of the dish (*n* = 7).

The viscosity of melted ice cream was measured using a sine-wave vibro viscometer (SV-10, A&D Company, Ltd., Tokyo, Japan) as described previously (Kumagai et al., 2026). A 10 mL aliquot of each sample was measured at 25 ± 2 °C, and the reading after 5 min was recorded as static viscosity (the product of viscosity and density; mPa·s·g/cm^3^) (*n* = 3).

#### 2.2.9 Melting resistance

The melting behavior and shape retention of the ice cream were evaluated by measuring the melting rate at room temperature (approximately 25 °C). Cube-shaped ice cream samples (3 cm × 3 cm × 3 cm) were placed on a wire mesh (6 mm opening) over a tared paper cup at room temperature, and the weight of dripped meltwater was recorded at 3–5 min intervals until complete melting. The melting rate was expressed as the percentage of melted liquid weight relative to the initial sample weight. Measurements were performed in quintuplicate (*n* = 5).

#### 2.2.10 Texture profile analysis (TPA)

TPA was conducted using a texture analyzer (RE2–33005, Yamaden Co., Ltd., Tokyo, Japan) equipped with a cylindrical flat-end plunger (10 mm diameter) at a penetration speed of 5 mm/s and a compression strain of 90%. A 20 N load cell was used as the standard setting; however, for samples with higher STC addition levels (0.3, 0.5, and 1.0%), a 200 N load cell was employed to accommodate the increased sample hardness and prevent the compression force from exceeding the instrument's capacity.

The following textural parameters were derived from the obtained force–time curves (Veer et al., 2019): hardness, adhesiveness, and gumminess. All measurements were performed in quintuplicate (*n* = 5) for each treatment, and mean values were used for subsequent analyses.

#### 2.2.11 FTIR analysis

ATR-IR spectra of freeze-dried ice cream samples (powder) and liquid samples collected from completely and partially thawed ice cream, both prepared at varying STC addition levels were recorded using an IRTracer-100 system (Shimadzu, Kyoto, Japan) equipped with an ATR accessory (MicroATR Vision, 100–14-0008, Czitek, Danbury, CT, USA). The spectra were acquired over the wavenumber range of 4000–400 cm^−1^ at a spectral resolution of 4 cm^−1^, and 80 scans were co-added for each spectrum. Two types of liquid samples were analyzed: (i) samples that had been completely thawed after freezing, and (ii) the liquid fractions collected from partially thawed samples after freezing. To correct for apparent spectral variations arising from differences in light scattering and contact conditions among samples, multiplicative scatter correction (MSC) was applied as a preprocessing step to the ATR-IR spectra of the powder samples. Second-derivative spectra were calculated using the Savitzky–Golay (SG) method with a second-order polynomial and a window size of 7 (15 data points in total), following smoothing under the same SG conditions. All spectral preprocessing procedures were performed using The Unscrambler X 10.3 (Camo Analytics, Oslo, Norway). Each sample was measured in triplicate, and the average spectrum was used for subsequent analysis.

#### 2.2.12 Enzyme-linked immunosorbent assay (ELISA)

To detect milk allergen content in the ice cream samples, FASPEK ELISA II® kits for casein and β-LG (Product Code: M2122, Morinaga Institute of Biological Science, Yokohama, Japan) were used according to the manufacturer's instructions (Total Milk ELISA Kit II, 2026). Briefly, 19 mL of extraction buffer was added to 1 g of each sample, and extraction was carried out by horizontal shaking at 20 °C for 20 h (EYELA, MMS-1020, Tokyo Rikakikai Co., Ltd., Tokyo, Japan). The resulting supernatant was diluted as necessary for analysis, and absorbance was measured at 450 nm (reference, 630 nm) using a microplate reader (SH-9000Lab, Corona Electric Co., Ltd.).

#### 2.2.13 Sensory evaluation

Sensory evaluation was conducted with an untrained panel of 64 students and faculty members at Shimane University, Japan. Five participants were excluded due to incomplete responses, and data from the remaining 59 participants (valid response rate: 92.2%; 7 males, 52 females) were used for analysis. The age distribution of the participants was as follows: teens (*n* = 23), twenties (*n* = 31), thirties (*n* = 1), forties (*n* = 2), fifties (*n* = 1), and sixties (*n* = 1). This study was conducted with approval from the Ethics Review Committee of the Faculty of Human Sciences, Shimane University (approval number: 231101–1). Written informed consent was obtained from all participants after full disclosure of the research objectives, voluntary nature of participation, and data anonymity. As an allergy precaution, all ingredients used, including milk, dairy products, eggs, and persimmon, were disclosed to participants prior to the study, and individuals with relevant allergies were excluded from participation.

Upon sample presentation, each ice cream sample was labeled with a randomly assigned three-digit code to blind the treatment groups. Samples were cut into approximately 1 cm cubes, and both the samples and serving spoons were kept in a freezer (−18 °C) until immediately before serving. Participants evaluated samples from all five treatment groups in a randomized order and rinsed their mouths with water between samples.

Each sample was evaluated on two dimensions—intensity and preference—using a 7-point Likert scale. Intensity attributes (color, aroma, astringency, smoothness, and meltability) were rated on a scale from −3 (weak) to +3 (strong). Preference attributes (appearance, aroma, flavor, texture, and overall evaluation) were rated on a scale from −3 (unfavorable) to +3 (favorable). The panelists were also free to offer comments on each sample.

#### 2.2.14 Statistical analysis

All statistical analyses were performed using SPSS (version 30; IBM, Armonk, NY, USA), including one-way analysis of variance (ANOVA) with eta squared (η^2^) as the effect size, followed by Tukey's test, and multiple linear regression (forced entry method) for sensory data. Differences were considered significant at *p* < 0.05. Microsoft Excel (Microsoft 365) was used to create figures and tables; only the sensory box plots were generated using the ggplot2, ggh4x, and tidyverse packages in R (version 4.5.0; R Foundation for Statistical Computing, Vienna, Austria).

## 3 Results and discussion

### 3.1 Selection of plant-derived tannin material

As described in Section 2.1.1, the 16 tannin sources (from 12 plant species) were selected in a two-step approach: approximately 300 plant materials were first screened for tannin content, and the selected sources were then evaluated for protein-binding capacity by SDS-PAGE of heated mixtures of each extract with whey protein and casein. The pH and STC of each extract were also measured (Fig. 1).

**Fig. 1:**
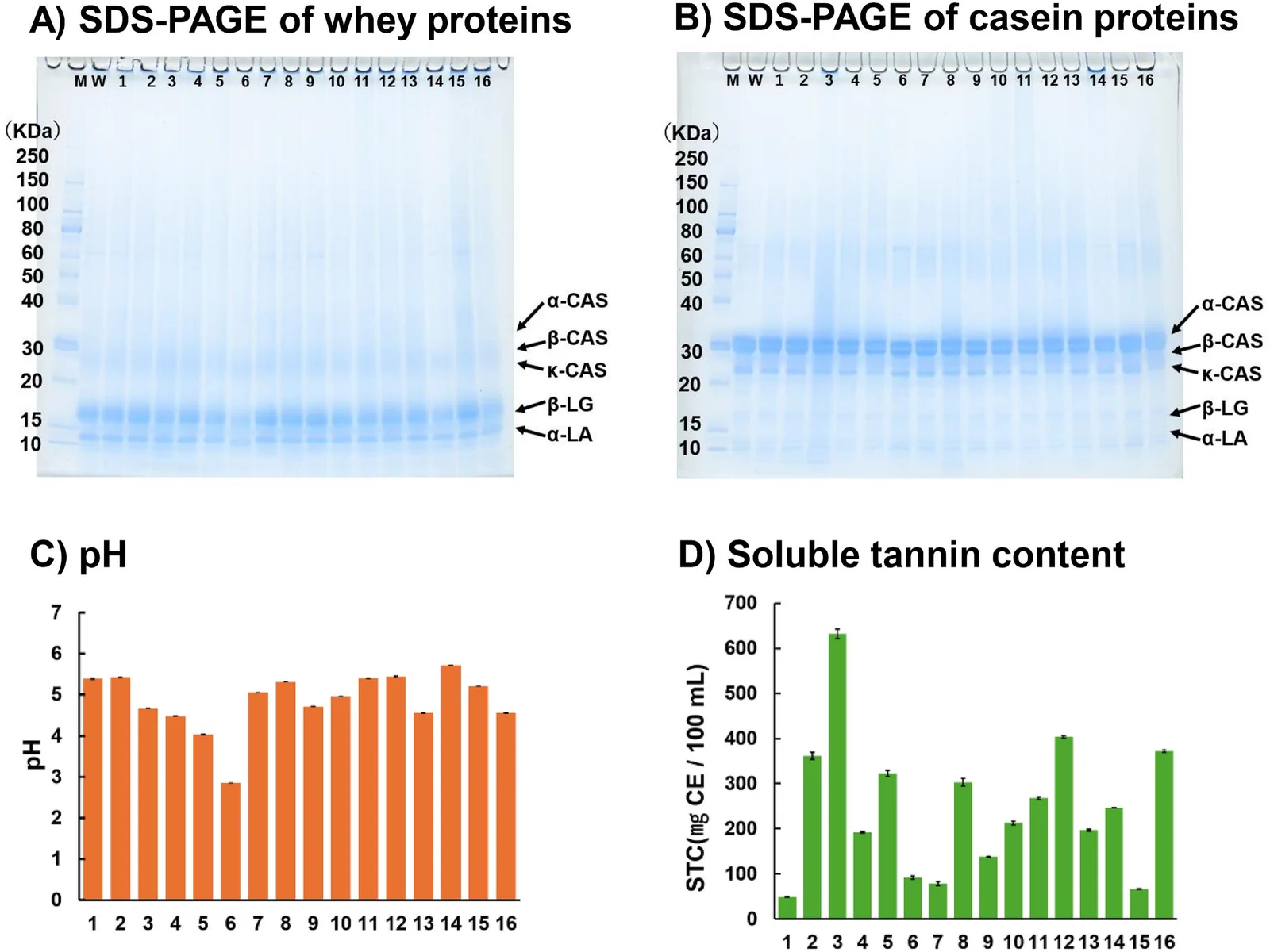
(A) SDS-PAGE of whey proteins, (B) SDS-PAGE of casein proteins, (C) pH, and (D) STC of the extracts themselves. Numbers 1–16 indicate the corresponding plant-derived tannin materials: 1: Ginkgo (*Ginkgo biloba*: fruits), 2: *Styrax obassia*, 3: Red bayberry (*Morella rubra*: buds), 4: Red bayberry (*Morella rubra*: young leaves), 5: Japanese chestnut ‘Porotan’ (*Castanea crenata* ‘Porotan’: immature burrs), 6: Grape ‘Shinku’ (*Vitis* ‘Shinku’: young fruits), 7: Silk tree (*Albizia julibrissin*: leaves), 8: Buddhist pine (*Podocarpus macrophyllus*: leaves), 9: White oak (*Quercus myrsinifolia*: young leaves), 10: White oak (*Quercus myrsinifolia*: mature leaves), 11: Oak (*Quercus serrata*: hulls), 12: Oak (*Quercus serrata*: fruits), 13: Persimmon (*Diospyros kaki*: calyxes), 14: Persimmon (*Diospyros kaki*: young fruits), 15: Chinese juniper (*Juniperus chinensis*: leaves), 16: Japanese mallotus (*Mallotus japonicus*: leaves). Abbreviations: STC, soluble tannin content; CE, catechin equivalent. (For interpretation of the references to color in this figure legend, the reader is referred to the web version of this article.)

As shown in Fig. 1A, the bands corresponding to whey proteins, β-LG and α-LA, were markedly attenuated in Lane 6 (young fruits of ‘Shinku’ grape), Lane 14 (young persimmon fruit), and Lane 16 (*Mallotus japonicus*) compared with the control (water, Lane W). Although this attenuation suggests tannin–protein complex formation, the extract in Lane 6 was strongly acidic (pH 2.9; Fig. 1C). Whey proteins are known to aggregate at pH below 3.0 by increasing structural flexibility and exposing and increasing surface hydrophobicity (Galm et al., 2017; Nicolai & Durand, 2013), and these aggregates are likely removed by centrifugation (Dissanayake et al., 2013). Therefore, the band attenuation in Lane 6 was attributed to acid-induced aggregation rather than tannin–protein interaction, and this material was excluded from further consideration.

As shown in Fig. 1D, Lane 3 (*Morella rubra* bud) exhibited the highest retained STC among the 16 materials, yet showed relatively little band attenuation (Fig. 1A). This suggests that a high retained STC does not necessarily correspond to strong protein-binding capacity, and that qualitative differences in tannin structure and degree of polymerization influence binding affinity (Gundale et al., 2010). Lane 14 (young persimmon fruit) contained approximately two-thirds of the polyphenol content of Lane 16 (*Mallotus japonicus*), yet showed comparable band attenuation, indicating superior binding efficiency per unit polyphenol content.

As shown in Fig. 1B, the bands corresponding to caseins (α-casein, β-casein, and κ-casein) showed only marginal attenuation in some lanes, with overall less pronounced changes compared with whey proteins. This is likely because casein forms micellar structures in which the hydrophobic core is sequestered internally (Gao et al., 2025), limiting the accessible surface area for tannin binding.

Based on these results, YPE was selected as the tannin material for subsequent experiments, as it demonstrated high binding affinity for whey proteins without interference from extreme acidity. Given that tannin binding to casein was limited, the present study focuses primarily on the effects of YPE addition on the physical properties of ice cream mediated through interactions with whey proteins, with milk allergen content evaluated as a secondary objective.

### 3.2 Physicochemical properties and functional characteristics of ice cream ingredients and products

The physicochemical properties of the dry mix solution (corresponding to the control) and YPE are shown in Fig. 2A. The dry mix solution exhibited high lightness (L* = 86.4), a slightly green hue (a* = −3.3), and a yellow hue (b* = 13.7), reflecting the milky-white appearance of dairy products, whereas YPE showed markedly lower L* (35.2) and a strong reddish tone (a* = 7.4).

**Fig. 2:**
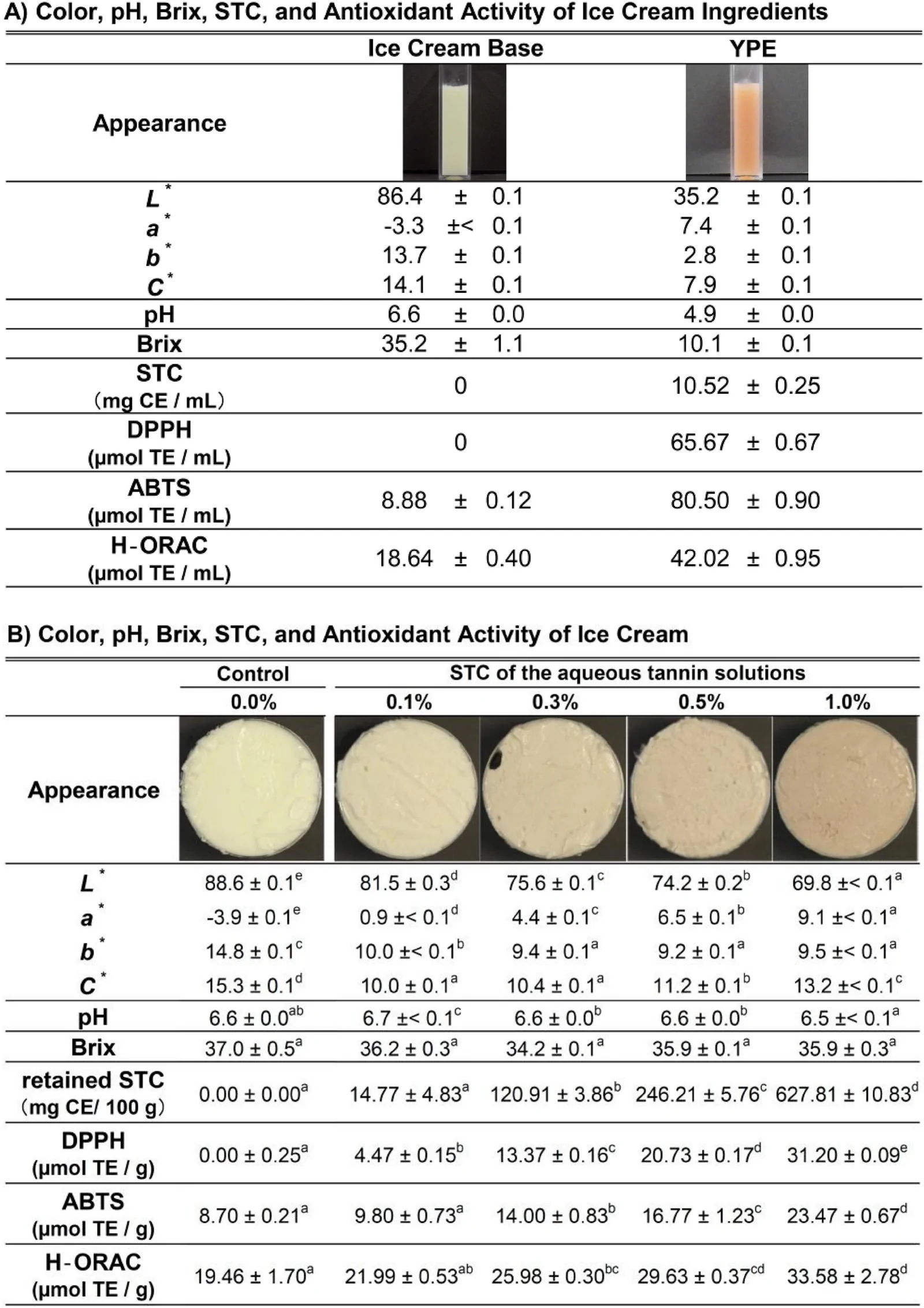
(A) Color, pH, Brix, STC, and antioxidant activity of ice cream ingredients (Ice cream base and YPE), and (B) Color, pH, Brix, retained STC, and antioxidant activity of ice cream prepared with varying concentrations of aqueous tannin solutions (0.0–1.0%). Photographs were taken under identical conditions without color or contrast adjustment (Section 2.2.3). Values represent the mean ± standard error (*n* = 5 for Color, pH and Brix, *n* = 6 for retained STC(B), DPPH(B), and ABTS, *n* = 4 for STC(A), H-ORAC, *n* = 3 for DPPH(A)). Different letters (a–e) within the same row indicate significant differences (*p* < 0.05). Abbreviations: YPE, young persimmon fruit extract; STC, soluble tannin content; CE, catechin equivalent; TE, Trolox equivalent; DPPH, 2,2-diphenyl-1-picrylhydrazyl; ABTS, 2,2′-azino-bis(3-ethylbenzothiazoline-6-sulfonic acid); H-ORAC, hydrophilic oxygen radical absorbance capacity.

The color and antioxidant properties of the ice cream are shown in Fig. 2B. With increasing STC addition levels, the L* value decreased from 88.6 in the control to 69.8 in the 1.0% added STC group, and the a* value shifted from negative (−3.9) to positive, reaching 9.1 at 1.0% added STC. These results confirm that the natural pigments derived from YPE (polyphenols) imparted a reddish-brown color without the use of artificial colorants.

The effects of YPE addition on pH and Brix were limited. Although YPE itself was acidic (pH 4.9), the pH of the ice cream remained within 6.5–6.7 across all groups (control, 6.6), likely owing to the strong buffering capacity of milk proteins and mineral salts. Similarly, because the Brix of YPE was low (10.1), its addition did not substantially alter the Brix of the ice cream (34.2–36.2 vs. 37.0 in the control), indicating that bioactive compounds can be incorporated without excessively increasing the soluble solids content or affecting freezing point depression.

Retained STC and antioxidant activity indices (DPPH, ABTS, and H-ORAC) all increased in a concentration-dependent manner with increasing STC addition levels. The retained STC of the 1.0% added STC ice cream was 627.81 mg CE/100 g. Given that YPE constituted 400 mL of the total 600 mL ice cream mix, the estimated tannin content attributable to YPE was approximately 941.72 mg CE/100 mL (627.81 × 3/2), which closely approximates the STC of the original YPE (1050 mg CE/100 mL). This agreement suggests that the tannins derived from YPE were largely retained in the final product and contributed substantially to its antioxidant capacity. DPPH radical scavenging activity increased most sharply, from 0.00 μmol TE/g in the control to 31.20 μmol TE/g in the 1.0% added STC group.

In summary, YPE addition imparted a concentration-dependent natural reddish-brown color to the ice cream without the use of artificial colorants, while its effects on pH and Brix remained limited due to the buffering capacity of the milk components and the low Brix of YPE. All antioxidant indices, including retained STC, DPPH, ABTS, and H-ORAC, increased with STC addition levels, with DPPH activity showing the most prominent increase, confirming that YPE addition effectively enhanced the antioxidant functionality of the ice cream.

### 3.3 Changes in microstructure and macroscopic physical properties

To elucidate the effects of YPE addition on the matrix structure of ice cream, freeze-dried samples were examined by SEM, the particle size distribution of the ice cream mix was measured, and the overrun, density, and viscosity of the products were determined.

SEM observation (Fig. 3A) revealed that the control exhibited a typical ice cream network structure consisting of relatively large, smooth, spherical voids—presumably corresponding to air bubble traces—surrounded by thin walls. With increasing STC addition levels, the voids became irregular in shape, and at 0.5 and 1.0% added STC, the walls became markedly thicker and denser, indicating a transition toward a more compact and aggregated matrix structure. Because SEM was performed on freeze-dried samples, the observed voids represent a proxy for, rather than a direct image of, the frozen structure; however, as all samples were processed identically, the relative differences among STC addition levels are considered to reflect treatment-induced changes.

**Fig. 3:**
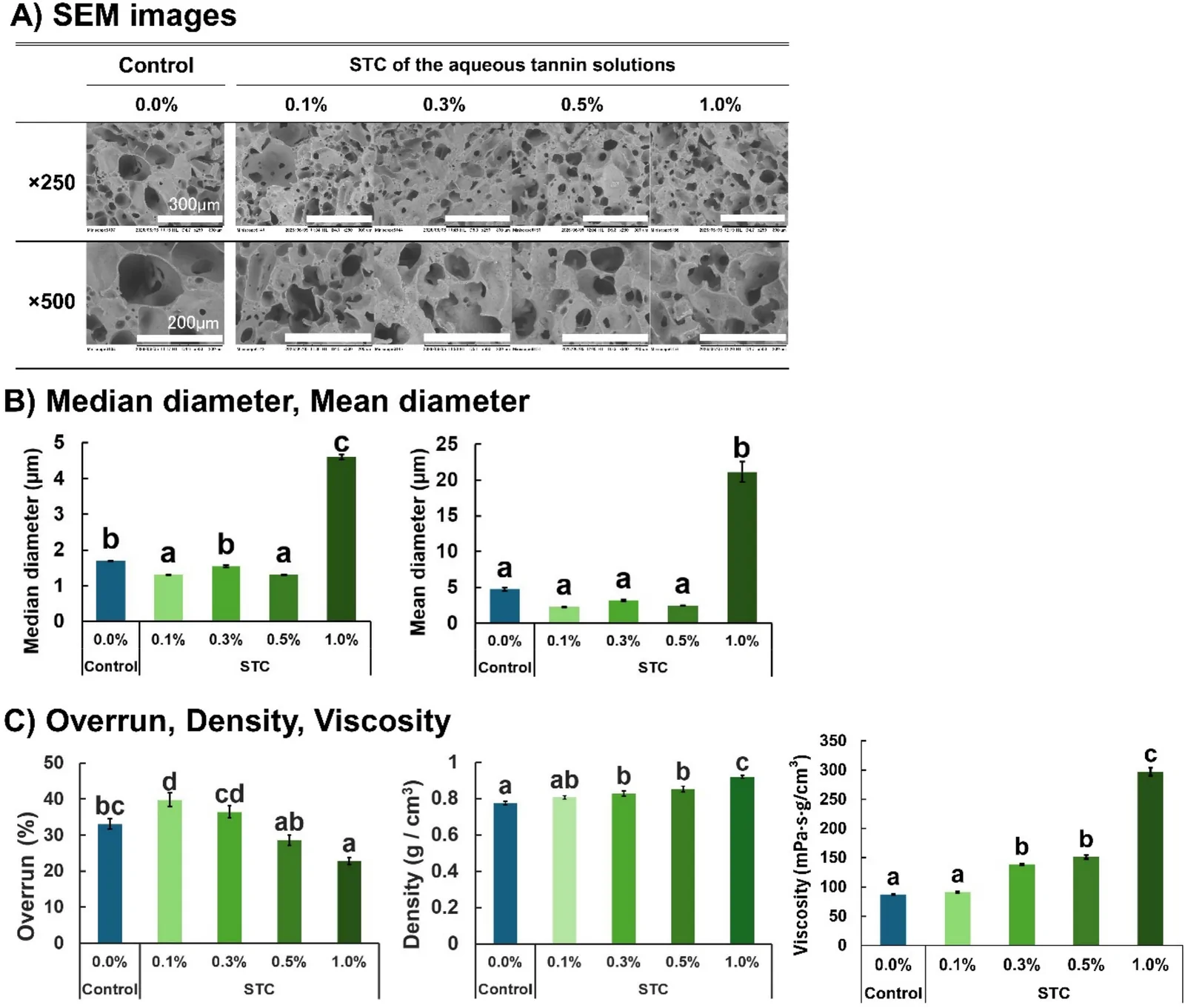
(A) SEM images of ice cream prepared with varying concentrations of aqueous tannin solutions (0.0–1.0%), (B) Median diameter and Mean diameter of ice cream prepared with varying concentrations of aqueous tannin solutions (0.0–1.0%), and (C) Overrun, density, and viscosity of ice cream prepared with varying concentrations of aqueous tannin solutions (0.0–1.0%). STC in (B) and (C) denotes the added STC level. Bars represent the mean ± standard error (*n* = 3 for Median diameter and Mean diameter, *n* = 5 for Overrun, *n* = 7 for Density, *n* = 3 for Viscosity). Different letters (a–d) indicate significant differences among the groups (*p* < 0.05). Abbreviations: STC, soluble tannin content.

These microstructural changes were corroborated by particle size distribution measurements (Fig. 3B). The mean particle diameter in the control was 4.75 μm, whereas it decreased to 2.25, 3.22, and 2.46 μm in the groups with 0.1, 0.3, and 0.5% added STC, respectively. This reduction suggests that polyphenols at moderate concentrations interact at the oil–water interface and transiently improve fat globule dispersibility (Farooq et al., 2021). In contrast, the mean particle diameter in the 1.0% added STC group increased markedly to 21.15 μm, approximately 4.5 times that of the control. This sharp increase, consistent with the wall thickening observed by SEM, suggests extensive cross-linking between high-concentration tannins and milk proteins, leading to the formation of large insoluble aggregates.

These microstructural changes were reflected in overrun, density, and viscosity (Fig. 3C). Overrun temporarily increased at 0.1% added STC (39.71%) compared with the control (33.03%), then decreased concentration-dependently to 22.70% at 1.0% added STC. Correspondingly, product density increased significantly from 0.78 g/cm^3^ in the control to 0.92 g/cm^3^ at 1.0% added STC, with no significant difference at 0.1% added STC. The static viscosity of melted ice cream did not differ between the control and 0.1% added STC (87.0 and 91.3 mPa·s·g/cm^3^), but increased significantly at 0.3 and 0.5% added STC (138.6 and 151.2 mPa·s·g/cm^3^) and reached 297.2 mPa·s·g/cm^3^ at 1.0% added STC, approximately 3.4 times that of the control.

The transient increase in overrun at 0.1% added STC, which occurred without an increase in viscosity, is likely related to the reduction in particle size, suggesting that polyphenols at low concentrations exhibit surfactant-like activity, promoting protein unfolding and foaming at the air–liquid interface (Xia et al., 2025). In contrast, the decrease in overrun and increase in density at higher STC addition levels coincided with the marked increase in viscosity caused by the dense matrix and large aggregates, because excessive viscosity of the ice cream mix inhibits air incorporation by the dasher (Pangastuti et al., 2025).

This concentration-dependent transition from a dispersing to an aggregating effect is consistent with the behavior reported for protein–polyphenol complexes, in which moderate polyphenol levels can enhance interfacial coverage and electrostatic or steric stabilization, whereas higher levels alter surface charge and promote aggregation (Li et al., 2021). In addition, bridging flocculation can occur when a single molecule or complex binds simultaneously to two or more droplet surfaces (Dickinson et al., 1989). These mechanisms support the interpretation that YPE tannins act in a surfactant-like, dispersing manner at low STC addition levels and in a bridging, cross-linking manner at 1.0% added STC. However, because surface tension and zeta potential were not measured in the present study, this interpretation remains to be confirmed, and such measurements are warranted in future work.

Taken together, the SEM observations, particle size distribution, overrun, density, and viscosity results are mutually consistent, indicating that high-concentration YPE addition (particularly at 1.0% added STC) induced a series of structural changes—matrix densification, large aggregate formation, increased viscosity, inhibition of air incorporation, and increased product density—likely mediated by cross-linking between tannins and milk proteins. These structural changes are considered the fundamental basis for the increased hardness and enhanced melting resistance described in the following sections.

### 3.4 Melting resistance and textural changes

Measurement of the melting behavior of ice cream at room temperature revealed a concentration-dependent improvement in melting resistance upon YPE addition (Fig. 4A). In the control, melting progressed rapidly, with complete melting (100.0%) at 42 min. In contrast, all YPE-added groups exhibited delayed melting; the 1.0% added STC group showed a melting rate of only 4.3% at 36 min and required approximately 75 min for complete melting—about 33 min longer than the control.

**Fig. 4:**
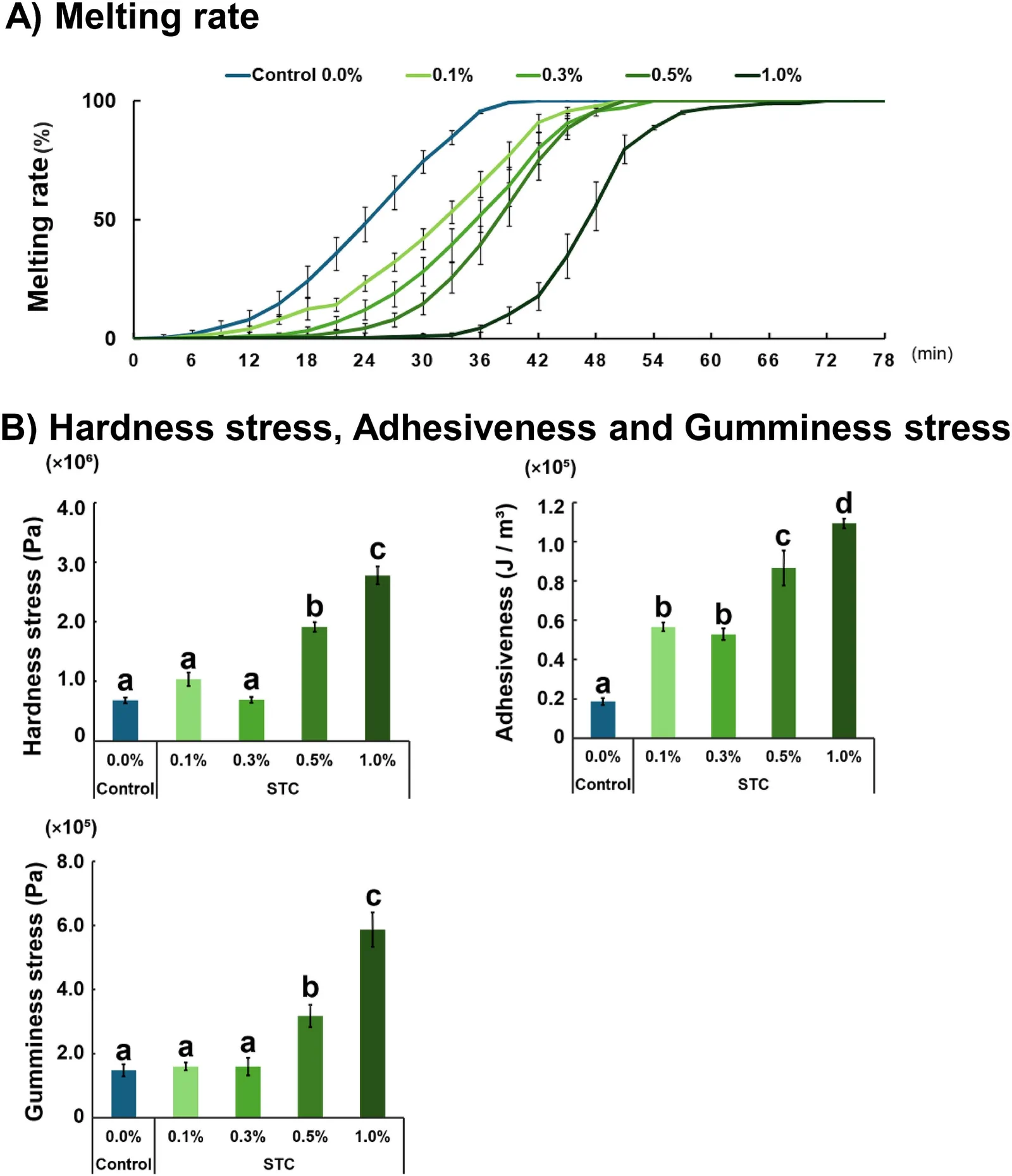
(A) Melting rate of ice cream prepared with varying concentrations of aqueous tannin solutions (0.0–1.0%), and (B) Hardness stress, Adhesiveness and Gumminess stress of ice cream prepared with varying concentrations of aqueous tannin solutions (0.0–1.0%). In (A), the plateau at 100% indicates complete melting of the sample; measurements were continued until no further dripping was observed. Bars represent the mean ± standard error (*n* = 5 for Melting rate, Hardness stress, Adhesiveness and Gumminess stress). Different letters (a–d) indicate significant differences among the groups (*p* < 0.05). Abbreviations: STC, soluble tannin content. STC denotes the STC addition level.

The melting behavior of ice cream is governed by multiple interacting factors, including ice crystal melting, the insulating effect of air bubbles, the aggregated fat globule network, and the water-holding capacity of the unfrozen serum phase (Freire et al., 2020). The high melting resistance of the YPE-added groups is consistent with the results in Section 3.3: the dense, highly viscous matrix formed through cross-linking between tannins and milk proteins likely inhibited the outflow of meltwater (Nehme et al., 2025), while the high-density structure resulting from reduced overrun increased resistance to gravity and prevented structural collapse of the matrix.

TPA (Fig. 4B) showed concentration-dependent increases in hardness (6.8 × 10^5^ to 2.8 × 10^6^ Pa), adhesiveness (1.9 × 10^4^ to 1.1 × 10^5^ J/m^3^), and gumminess (1.5 × 10^5^ to 5.9 × 10^5^ Pa) from the control to the 1.0% added STC group, with significant changes in hardness and gumminess at 0.5% added STC and above. These textural changes are consistent with the matrix densification and large aggregate formation described in Section 3.3 and likely reflect tannin–milk protein cross-linking at the bulk mechanical level (Sapuła et al., 2023).

Taken together, these results suggest that tannins in YPE cross-link with milk proteins, densifying the ice cream matrix and increasing its viscosity, which likely inhibits air incorporation, reduces overrun, and increases product density. This high-density, highly adhesive matrix likely suppresses meltwater outflow and prevents structural collapse, resulting in markedly enhanced melting resistance.

These physical changes may also add value as product characteristics: the increased hardness and adhesiveness correspond to a rich, gelato-like mouthfeel, and the enhanced melting resistance offers practical advantages for quality retention during distribution and consumption. Furthermore, the simultaneous achievement of natural reddish-brown coloration and matrix stabilization without chemical stabilizers is meaningful in the context of the clean-label trend. However, these functional benefits at 1.0% added STC must be weighed against consumer acceptability. The marked increases in hardness, adhesiveness, and gumminess at this level may have contributed to the significant decline in overall evaluation observed at 1.0% added STC (Section 3.6), whereas overall evaluation remained comparable to the control up to 0.5% added STC. Considering that 0.1–0.5% added STC still extended the complete melting time from 42 min in the control to 51–54 min and significantly increased antioxidant activity (DPPH at all levels; ABTS and H-ORAC at 0.3% added STC and above; Fig. 2), this range is considered more practical for balancing functional performance with consumer acceptability within the present panel.

### 3.5 FTIR analysis

Fig. 5A shows the ATR-IR spectra of the freeze-dried ice cream samples (powder) with varying STC addition levels. Several IR bands were observed at 1743, 1650, 1540, 1450–1200, 1038, 990, and 927 cm^−1^. Since these bands were present in the control (0.0%) sample, they were attributed to proteins, lipids, and saccharides derived from the ice cream ingredients. The band at 1743 cm^−1^ was assigned to the C=O stretching vibration of lipids and saccharides, while the bands at 1650 and 1540 cm^−1^ were assigned to the amide I and amide II bands of proteins, respectively (Barth, 2007; Dong et al., 1990). The broad region between 1450 and 1200 cm^−1^ was attributed to CH₃/CH₂ deformation, twisting, and bending vibrations of lipids, proteins, and saccharides, together with the amide III band of proteins. The bands at 1038 and 990 cm^−1^ were assigned to C—H, C—C, and C—N stretching vibrations, whereas the band at 927 cm^−1^ was attributed to the C—C stretching vibration of proteins.

**Fig. 5:**
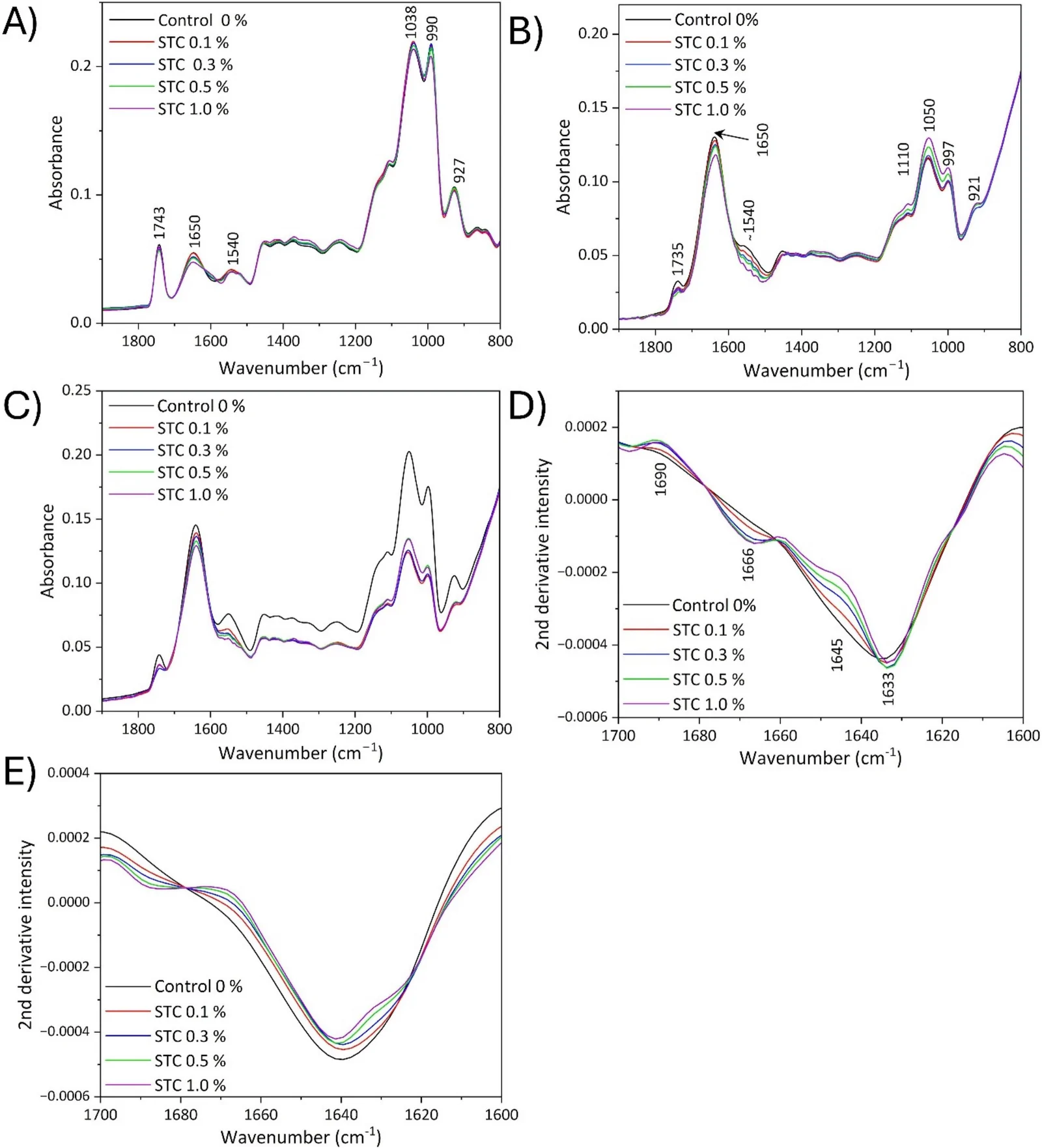
ATR-IR spectra in the 1900–800 cm^−1^ region of (A) freeze-dried ice cream samples (powder), (B) liquid samples collected from completely thawed ice cream, and (C) liquid samples collected from partially thawed ice cream. Second-derivative spectra in the 1700–1600 cm^−1^ region of (D) completely thawed samples and (E) partially thawed samples. Abbreviations: STC, soluble tannin content. STC denotes the STC addition level.

For the liquid samples, the spectra shown in Fig. 5B were obtained from samples that had been completely thawed after freezing. In the aqueous samples, the spectra exhibited bands at similar wavenumbers to those of the powder samples (Fig. 5A), even though slight shifts in band positions were observed. Notably, the band at approximately 1650 cm^−1^ was more intense in the aqueous samples. This band can be assigned to the H–O–H bending vibration of water (Maréchal, 2011), which overlaps with the amide I band of proteins. With increasing STC addition levels, the intensity of the band at 1540 cm^−1^ decreased, whereas the band intensity in the 1400–900 cm^−1^ region increased. These results indicate that the bands in the 1400–900 cm^−1^ region originate from YPE. This observation is consistent with a previous study, in which persimmon paste exhibited characteristic bands in the same spectral region (Tsurunaga et al., 2022). Thus, the ATR-IR spectra of the liquid samples clearly capture the concentration-dependent changes in STC addition. On the other hand, the band at 1540 cm^−1^ is attributed to ice cream ingredients, suggesting that its intensity should slightly decrease with increasing STC addition. However, the almost complete disappearance of this band at 1.0% added STC cannot be readily explained by simple dilution effects alone. The decrease in the amide II band observed in the presence of tannins has been widely reported and is often attributed to interactions between tannins and proteins. Specifically, such interactions can restrict the freedom of N—H groups involved in amide bonds, leading to a decrease in the intensity of the amide II band. In addition, tannin–protein interactions may induce conformational changes in proteins. Indeed, examination of the amide I region revealed a systematic shift of the amide I band from 1640 to 1633 cm^−1^ with increasing STC addition. To further examine this issue, second-derivative spectra were calculated in the region around 1650 cm^−1^ (Fig. 5D). The results showed that, in addition to the contribution from the water band at 1633 cm^−1^, three second-derivative peaks attributable to the amide I band exhibited changes in intensity with increasing STC addition levels. The peak at 1666 cm^−1^ was assigned to α-helix structures, whereas the peaks at 1690 and 1645 cm^−1^ were assigned to β-sheet structures of proteins (Barth, 2007; Dong et al., 1990). Based on the changes in the second-derivative peak intensities, the proportion of β-sheet structures appears to decrease, whereas that of α-helix structures appears to increase with increasing STC addition levels. These results suggest that interactions between tannins and milk proteins induce conformational changes in the proteins, which are reflected in both the shift of the amide I band and the decrease in the intensity of the amide II band (Edelmann & Lendl, 2002; Ricci et al., 2015).

Fig. 5C shows the ATR-IR spectra of the liquid fractions collected from partially thawed ice cream samples. For the liquid fraction collected from the partially thawed control sample (0.0%), solute-derived bands were markedly more intense than those observed in the corresponding completely thawed sample (Fig. 5B), indicating that the liquid fraction released during the initial stage of melting contained a relatively high concentration of dissolved components. These results suggest that the control ice cream melted with a concentration gradient, in which a concentrated liquid phase was released first, followed by fractions containing lower concentrations of dissolved solutes. This pattern is consistent with the freeze-concentration behavior of conventional ice cream, in which solutes become concentrated in the unfrozen serum phase during freezing (Goff, 2002; Muse & Hartel, 2004). In contrast, for the YPE-added samples, the intensities of the solute-derived bands in the partially thawed liquid fractions were comparable to those in the completely thawed samples, indicating that the solute concentrations were nearly identical. This departure from the concentration-gradient pattern typically observed in conventional ice cream (Goff, 2002; Muse & Hartel, 2004) suggests that STC addition altered the classical freeze-concentration-driven melting behavior, resulting in a more homogeneous release of dissolved components without an apparent concentration gradient. To our knowledge, such an effect of tannin addition on solute distribution during melting has not been previously reported; however, the underlying mechanism remains to be confirmed. Furthermore, with increasing STC addition levels, the intensities of the solute-derived bands in the partially thawed liquid fractions tended to decrease slightly, suggesting that melting began with a somewhat more dilute liquid phase at higher STC addition levels, whereas the opposite trend was observed in the completely thawed samples. These observations indicate that YPE alters the melting behavior of ice cream.

In addition, the partially thawed liquid fractions from YPE-treated samples exhibited a markedly stronger band at approximately 1540 cm^−1^, which increased with increasing STC addition levels, compared with the corresponding completely thawed samples. The band at approximately 1650 cm^−1^ also shifted to higher wavenumbers with increasing STC addition levels. These spectral features indicate that the protein structure in the liquid released during the early stage of melting differs from that in the completely thawed samples and more closely resembles that in the samples without added STC. This interpretation is further supported by comparing the second-derivative spectra of the partially thawed liquid fractions shown in Fig. 5E with those of the completely thawed samples shown in Fig. 5D. The results therefore suggest that YPE-induced changes in the conformation of milk proteins may be associated with the improved melting resistance of ice cream, although a quantitative relationship with melting and textural parameters remains to be established.

### 3.6 Consumer acceptability and determination of optimal addition concentration

Box plots for sensory attributes that differed significantly among treatment groups are shown in Fig. 6. No significant differences were observed among treatment groups for meltability intensity, texture preference, aroma intensity, or aroma preference (Supplementary Fig. S1). All mean values ± standard error, as well as the results of the significance tests, are shown in Supplementary Table S1.

**Fig. 6:**
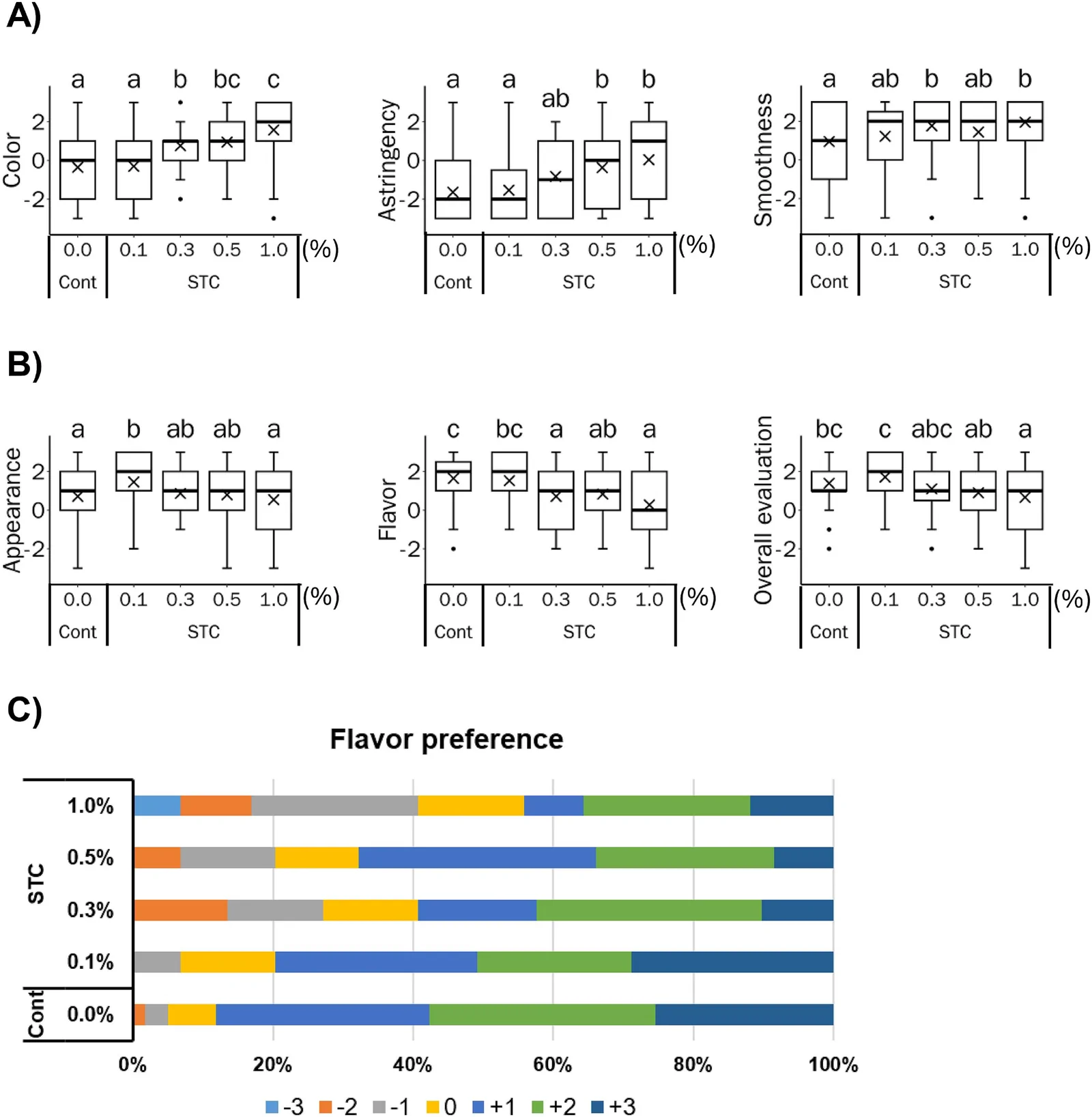
Significant differences were observed in six of the ten sensory attributes evaluated (*p* < 0.05). (A) Box plots for the intensity scales (Color, Astringency, Smoothness) of ice cream prepared with varying concentrations of aqueous tannin solutions (0.0–1.0%), (B) Box plots for the preference scales (Appearance, Flavor) and overall evaluation of ice cream prepared with varying concentrations of aqueous tannin solutions (0.0–1.0%), and (C) Proportion of preference scores for flavor preference (*n* = 59). X marks indicate mean values; bold lines indicate medians; box edges represent the interquartile range; whiskers extend to the most extreme values within 1.5 × interquartile range; and dots represent outliers (*n* = 59). Different letters (a–c) indicate significant differences among the groups (*p* < 0.05). Abbreviations: STC, soluble tannin content; Cont, control. STC denotes the STC addition level.

Smoothness tended to increase with STC addition levels and was lowest in the control. Astringency intensity showed no significant difference from the control at STC addition levels up to 0.3% but increased significantly at 0.5% added STC and above. Color intensity increased in a stepwise manner with increasing STC addition levels.

Flavor preference showed no significant difference from the control at 0.1% added STC but tended to decrease with increasing STC addition levels. Appearance preference was significantly higher in the 0.1% added STC group than in the control, while all other groups were comparable to the control. Overall evaluation was highest in the 0.1% added STC group, with no significant differences observed from the control through 0.5% added STC, whereas the 1.0% added STC group showed a significant decline. The effect of the STC addition level was significant for flavor preference (η^2^ = 0.113), overall evaluation (η^2^ = 0.069), and appearance preference (η^2^ = 0.044), whereas large inter-individual variability remained (Fig. 6C).

Lactones present in milk fat are known to impart coconut- and peach-like aromas (Schlutt et al., 2007), while tannins have been reported to possess deodorizing activity against malodorous compounds such as ammonia (Sepperer et al., 2020). The absence of aroma changes may be attributable to the lipophilic nature of lactones remaining dissolved in milk fat (Schlutt et al., 2007), suppression of volatile perception at low temperature, and the inherently low aroma intensity of the ice cream base.

Although differences in melting behavior and the increases in hardness, adhesiveness, and gumminess observed by TPA were expected to influence meltability intensity and texture preference, no significant changes were observed in either attribute, whereas smoothness intensity was significantly higher in the high-concentration groups. At low STC addition levels, the light, airy texture likely produced a soft, rapidly dissolving mouthfeel, whereas at high STC addition levels, a moderately firm yet smooth, lingering mouthfeel was likely generated. Stabilizer addition has been reported to increase the richness and smoothness of ice cream (Zaini et al., 2023), and this shift in the nature of the mouthfeel may account for the absence of significant differences in meltability intensity and texture preference.

With the addition of YPE, color intensity increased, whereas lightness (L*) decreased. Appearance preference generally decreased from 0.1 to 1.0% added STC, with the control ranking just above the 1.0% added STC group, consistent with the lower chroma (C*) values. These results suggest that the perception of color intensity was primarily driven by decreased lightness, and that the highest appearance preference at 0.1% added STC reflects a potential consumer preference for color tones with low chroma and high lightness.

Flavor preference was high in the control and 0.1% added STC groups and tended to decrease at higher STC addition levels. However, preference did not uniformly decrease across all panelists; a certain proportion of panelist assigned high scores even to the 0.5% and 1.0% added STC groups (Fig. 6C). Astringency intensity tended to increase with YPE addition (Fig. 6A), and its impact on preference should be considered when selecting the addition level within the practical range of 0.1–0.5% added STC. Nevertheless, some panelists expressed a preference for the 1.0% added STC group despite its stronger astringency. The free comments on the samples with higher STC addition levels also included several positive evaluations, such as “It tasted sweeter,” “It felt softer,” and “It was rich and smooth” (translated from Japanese). These results suggest that formulations with higher STC addition levels may be more appealing than the control to some consumers, and that these attributes could be leveraged in future product development.

Multiple regression analysis with overall evaluation as the dependent variable and preferences for appearance, aroma, flavor, and texture as independent variables yielded an *R*^2^ value of 0.670 and an adjusted *R*^2^ value of 0.666. The standardized regression coefficients (β) were statistically significant (all *p* < 0.05) in descending order: flavor (0.641), texture (0.217), appearance (0.146), and aroma (0.087). Although statistically significant, the aroma coefficient was smaller than those of the other three variables, indicating a comparatively limited practical contribution to overall evaluation. All variance inflation factor (VIF) values were low (1.075–1.220), indicating no multicollinearity concerns. Although flavor preference, the strongest predictor, decreased significantly at 0.3% added STC and above, overall evaluation did not decrease significantly up to 0.5% added STC. This is likely because aroma, texture, and appearance preference, as well as factors not explained by the model, decreased less than flavor preference or were maintained or increased at 0.5% added STC. The fact that smoothness and texture preference did not decline—and smoothness even tended to improve—at high STC addition levels, despite the hardening indicated by TPA, is considered an advantage of YPE addition. Future studies could also focus on attributes other than flavor that improve acceptability.

A limitation of this study concerns the sensory panel. As described in Section 2.2.13, the panel was predominantly composed of young, university-affiliated individuals (52 women and 7 men among 59 valid respondents; 54 of 59 aged 18–29), rather than a demographically balanced sample of the general consumer population. Sensory perception and preference—particularly for astringency, sweetness, and overall flavor—vary with age, sex, and dietary habits (Bhuiyan, 2026), and sensitivity to astringency has been reported to change with age (Wang et al., 2022). Consumers in other demographic groups may therefore respond differently to YPE-containing ice cream, and the acceptable range of STC addition levels may differ, particularly among age groups. Accordingly, the acceptability trends reported here, including the favorable evaluation of 0.1–0.5% added STC, should be interpreted as reflecting the preferences of this specific panel, and further studies involving a broader, demographically balanced consumer panel are warranted.

### 3.7 Effects of YPE addition on milk allergen content under different extraction conditions

To evaluate the effects of YPE addition on milk allergen content, ice cream extracts prepared under different extraction and simulated digestion conditions (ELISA extraction buffer, phosphate buffer, and oral, gastric, and intestinal phases) were analyzed by SDS-PAGE (Fig. 7A) and sandwich ELISA (Fig. 7B).

**Fig. 7:**
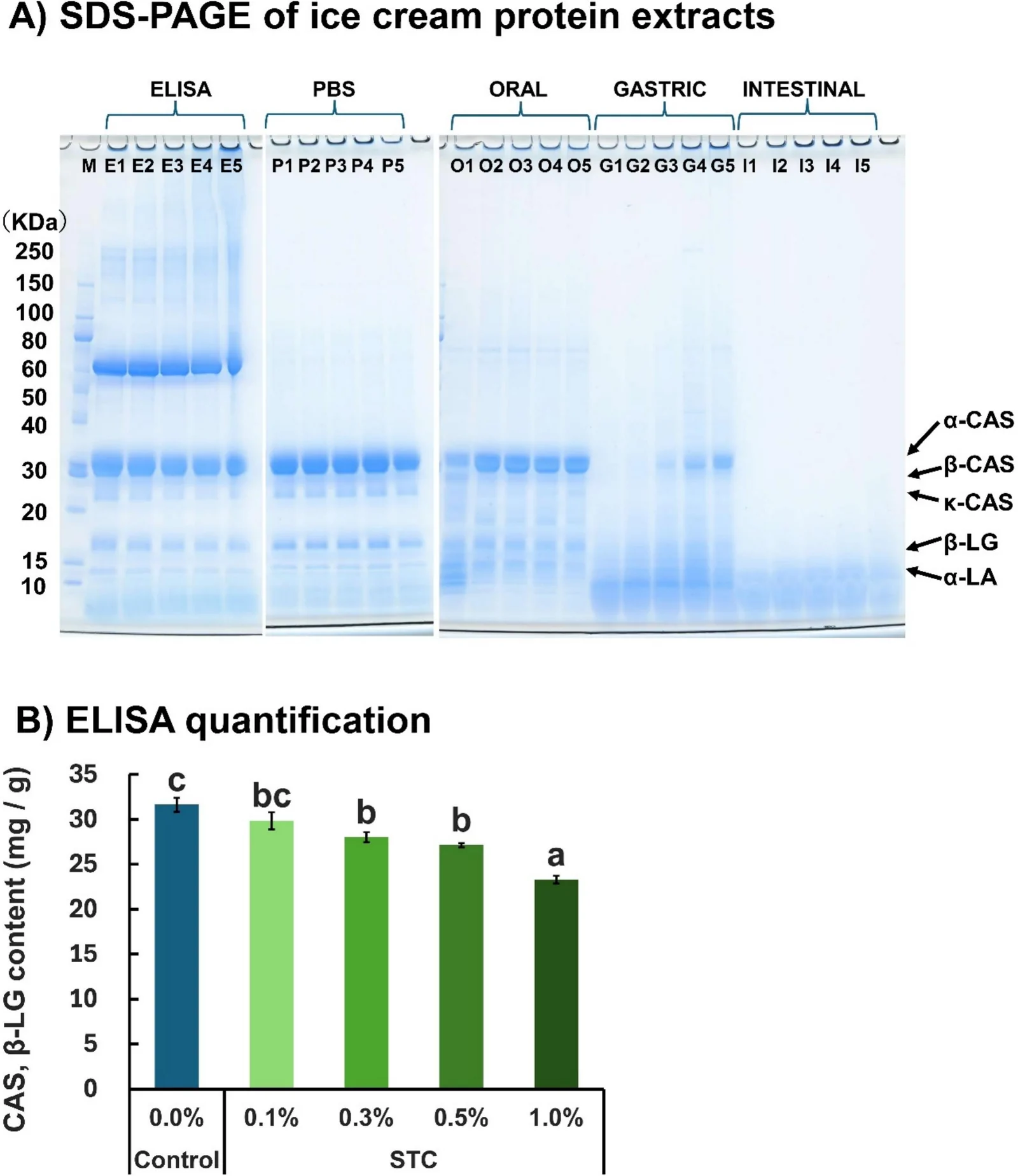
(A) SDS-PAGE of ice cream protein extracts treated with ELISA buffer, phosphate buffer, and simulated digestion phases (oral, gastric, and intestinal). In the lane labels, the initial capital letter indicates the treatment condition (E, ELISA; P, phosphate buffer; O, oral; G, gastric; I, intestinal), and the subsequent numbers (1–5) represent the treatment groups as follows: 1, Control-0.0%; 2, STC-0.1%; 3, STC-0.3%; 4, STC-0.5%; and 5, STC-1.0%, and (B) Milk allergen protein (CAS, β-LG) content, quantified by sandwich FASPEK ELISA II® kit. Bars represent the mean ± standard error (*n* = 4 for Milk allergen protein content, quantified by sandwich ELISA). Different letters (a–c) indicate significant differences among the groups (*p* < 0.05). Abbreviations: CAS, casein; β-LG, β-lactoglobulin; STC, soluble tannin content; Cont, control. STC denotes the STC addition level.

SDS-PAGE results (Fig. 7A) showed that during the oral and particularly gastric phases, bands corresponding to α-casein, β-casein, κ-casein, β-LG, and α-LA tended to be more intense in groups with higher STC addition levels. This suggests that the presence of YPE delays enzymatic degradation and enhances protein stability, especially under the acidic conditions of the gastric phase. In the intestinal phase, bands corresponding to allergen proteins were markedly attenuated across all treatment groups, confirming the overall functionality and efficacy of the simulated digestion system.

ELISA quantification (Fig. 7B) showed that the casein and β-LG content decreased with increasing STC addition levels, from 31.6 mg/g in the control to 29.9, 28.0, 27.2, and 23.3 mg/g at 0.1, 0.3, 0.5, and 1.0% added STC, respectively. The decreases were significant at 0.3% added STC and above, with the largest reduction (approximately 26%) at 1.0% added STC.

Taken together, YPE addition reduced the ELISA-detectable milk allergen content in a concentration-dependent manner; however, the reduction was modest, and allergen proteins remained clearly detectable at all STC addition levels. Compared with reports on plant proteins in which polyphenol addition reduced allergen detection using similar SDS-PAGE and ELISA platforms (Qiu et al., 2025), the effect observed here was limited. Several factors may account for this difference. First, such studies often employed alkaline conditions (e.g., pH shifting) that promote polyphenol oxidation to quinones and covalent protein conjugation, whereas YPE was added under near-neutral conditions, under which tannin–protein interactions are expected to be predominantly non-covalent and potentially reversible during sample extraction. Second, those studies used isolated proteins (e.g., soy protein isolate) in model systems, whereas the present study used a complex dairy matrix in which caseins form stable micelles. The densely arranged surface layer of these micelles creates a steric barrier, which may limit tannin access to internal epitopes (Gao et al., 2025). Third, the autoclaving step used to prepare YPE (120 °C, 20 min) may have altered the degree of polymerization of persimmon tannins, as reported for thermal processing of other tannin-rich materials (Chamorro et al., 2012; González et al., 2022), potentially reducing their protein-binding capacity; direct characterization of tannin polymerization before and after autoclaving is warranted in future work. It should also be noted that SDS-PAGE and sandwich ELISA evaluate protein profiles and antibody-detectable antigen levels, respectively, and do not directly reflect IgE-mediated allergenicity. Changes in ELISA signal may arise from epitope masking, altered extractability of tannin–protein complexes, or interference with antibody binding. Therefore, the present data do not permit conclusions regarding changes in clinical allergenicity in either direction; IgE-binding assays using sera from patients with cow's milk allergy and cell-based assays (e.g., basophil activation tests) are required for such evaluation. In addition, only casein and β-LG were quantified in the present study; other milk allergens, such as α-LA, bovine serum albumin, lactoferrin, and immunoglobulins (Villa et al., 2018), should also be evaluated in future studies.

## 4 Conclusion

The present study demonstrated that YPE, a tannin-rich by-product of fruit-thinning operations, modified the physical properties and antioxidant functionality of ice cream in an added STC-dependent manner. SDS-PAGE screening of 16 plant-derived tannin materials identified YPE as an effective binder of whey proteins (β-LG and α-LA). In ice cream, YPE addition was associated with matrix densification, increased viscosity, reduced overrun, and increased product density, which are likely attributable to tannin–milk protein interactions. These changes improved melting resistance—complete melting was delayed from 42 min in the control to 51–54 min at 0.1–0.5% (peaking at 0.3%) added STC and to 75 min at 1.0% added STC—and increased hardness, adhesiveness, and gumminess in an added STC-dependent manner. FTIR analysis suggested that solutes were released more homogeneously during melting in YPE-added ice cream than in the control. YPE also imparted a natural reddish-brown color and significantly increased antioxidant activity without artificial colorants or chemical stabilizers. Overall acceptability was maintained up to 0.5% added STC, with 0.1% added STC receiving the highest scores, but declined at 1.0% added STC; thus, 0.1–0.5% added STC represents a practical range for balancing functional performance and consumer acceptability. As an exploratory objective, milk allergen content was also evaluated; sandwich ELISA showed a modest, concentration-dependent reduction in detectable casein and β-LG content (up to approximately 26%), although effects on clinical allergenicity remain to be determined. Taken together, YPE is a promising clean-label, multifunctional additive for ice cream whose value lies in melting resistance, texture modification, antioxidant enrichment, and natural coloration, while also adding value to an otherwise discarded agricultural by-product.

## Studies in Human

This study was performed in compliance with relevant laws, regulatory frameworks and guidelines where the research took place.

This study was approved by the Committee of the Faculty of Human Sciences, Shimane University.

(Approval No. 231101–1)

## CRediT authorship contribution statement

**Miyuna Kobayashi:** Writing – review & editing, Writing – original draft, Visualization, Validation, Investigation, Formal analysis, Data curation. **Tomoyoshi Hara:** Writing – original draft, Visualization, Validation, Investigation, Formal analysis, Data curation. **Akari Sunahara:** Writing – original draft, Investigation. **Mika Ishigaki:** Writing – original draft, Visualization, Validation, Methodology, Formal analysis. **Yoko Tsurunaga:** Writing – review & editing, Writing – original draft, Validation, Supervision, Resources, Project administration, Methodology, Funding acquisition, Formal analysis, Data curation, Conceptualization.

## Informed consent and patient details

Written informed consent to take part in the study and to publish the article has been obtained from all participants or their legal representatives. The privacy rights of participants have been observed.

## Declaration of generative AI and AI-assisted technologies in the writing process

The authors used generative AI tools (Gemini, Copilot, and Claude) to assist with grammar improvement, language editing, sentence structure, and text optimization. All AI-assisted content was carefully reviewed, verified, and edited by the authors. The authors take full responsibility for all publication content.

## Funding

This study was funded by the 10.13039/100017103Urakami Foundation for Food and Food Culture Promotion, SDGs Research Project of Shimane University, and JSPS KAKENHI (Grant Number JP23K20667).

## Declaration of competing interest

The authors declare the following financial interests/personal relationships which may be considered as potential competing interests: Yoko Tsurunaga reports financial support was provided by the Urakami Foundation for Food and Food Culture Promotion. Yoko Tsurunaga reports financial support was provided by JSPS. If there are other authors, they declare that they have no known competing financial interests or personal relationships that could have appeared to influence the work reported in this paper.

## Data Availability

Data will be made available on request.
