# Supplementary material for: Young persimmon fruit extract as a multifunctional natural additive in ice cream: Effects on physical properties, antioxidant functionality, and sensory acceptability
Source: Food Chem X. 2026 Oct 23;40:104662. doi: 10.1016/j.fochx.2026.104662 (PMC13722081; doi:10.1016/j.fochx.2026.104662)
Supplement: Supplementary file 1 [file mmc1.docx]

**Supplementary Fig. S1**

|  |  |  | Cont 0.0% | STC 0.1% | STC 0.3% | STC 0.5% | STC 1.0% |
| --- | --- | --- | --- | --- | --- | --- | --- |
| Intensity scales | Color | Mean | -0.36 | -0.31 | 0.76 | 0.95 | 1.58 |
|  |  | Standard error | ± 0.23 | ± 0.24 | ± 0.14 | ± 0.16 | ± 0.19 |
|  |  | Significant difference | a | a | b | bc | c |
|  | Aroma | Mean | -0.14 | -0.08 | -0.41 | -0.49 | -0.34 |
|  |  | Standard error | ± 0.22 | ± 0.20 | ± 0.21 | ± 0.20 | ± 0.20 |
|  |  | Significant difference | a | a | a | a | a |
|  | Astringency | Mean | -1.64 | -1.54 | -0.83 | -0.37 | 0.03 |
|  |  | Standard error | ± 0.21 | ± 0.21 | ± 0.24 | ± 0.24 | ± 0.27 |
|  |  | Significant difference | a | a | ab | b | b |
|  | Smoothness | Mean | 0.95 | 1.22 | 1.76 | 1.44 | 1.95 |
|  |  | Standard error | ± 0.24 | ± 0.22 | ± 0.17 | ± 0.20 | ± 0.18 |
|  |  | Significant difference | a | ab | b | ab | b |
|  | Meltability | Mean | 1.41 | 1.51 | 1.32 | 1.00 | 1.03 |
|  |  | Standard error | ± 0.21 | ± 0.19 | ± 0.19 | ± 0.21 | ± 0.23 |
|  |  | Significant difference | a | a | a | a | a |
| Preference scales | Appearance | Mean | 0.71 | 1.46 | 0.86 | 0.80 | 0.54 |
|  |  | Standard error | ± 0.22 | ± 0.18 | ± 0.16 | ± 0.18 | ± 0.21 |
|  |  | Significant difference | a | b | ab | ab | a |
|  | Aroma | Mean | 0.64 | 0.66 | 0.39 | 0.29 | 0.24 |
|  |  | Standard error | ± 0.17 | ± 0.15 | ± 0.13 | ± 0.14 | ± 0.16 |
|  |  | Significant difference | a | a | a | a | a |
|  | Flavor | Mean | 1.64 | 1.53 | 0.71 | 0.83 | 0.27 |
|  |  | Standard error | ± 0.15 | ± 0.16 | ± 0.21 | ± 0.18 | ± 0.24 |
|  |  | Significant difference | c | bc | a | ab | a |
|  | Texture | Mean | 1.39 | 1.76 | 1.46 | 1.42 | 1.46 |
|  |  | Standard error | ± 0.18 | ± 0.15 | ± 0.17 | ± 0.17 | ± 0.20 |
|  |  | Significant difference | a | a | a | a | a |
|  | Overall evaluation | Mean | 1.39 | 1.71 | 1.10 | 0.90 | 0.66 |
|  |  | Standard error | ± 0.16 | ± 0.15 | ± 0.18 | ± 0.17 | ± 0.22 |
|  |  | Significant difference | bc | c | abc | ab | a |

Supplementary Table S1

Supplementary Fig. S1 The four sensory attributes without significant differences and the proportion of preference scores for the characteristic attribute.

(A) Box plots for the intensity scales (Aroma, Meltability) of ice cream prepared with varying concentrations of aqueous tannin solutions (0.0–1.0%), (B) Box plots for the preference scales (Aroma, Texture) and overall evaluation of ice cream prepared with varying concentrations of aqueous tannin solutions (0.0–1.0%). (C) Proportion of preference scores for overall evaluation (*n* = 59).
X marks indicate mean values; bold lines indicate medians; box edges represent the interquartile range; whiskers extend to the most extreme values within 1.5 × interquartile range; and dots represent outliers (*n* = 59). All data labels are marked with "a", indicating no significant difference (p ≥ 0.05).

Abbreviations: Cont, control; STC, soluble tannin content of the aqueous tannin solutions. STC denotes the STC addition level.

Supplementary Table S1 Sensory evaluation scores of ice cream prepared with varying concentrations of aqueous tannin solutions.

Values represent the mean ± standard error (*n* = 59). Different letters (a–c) within the same row indicate significant differences (*p* < 0.05).

Abbreviations: Cont, control; STC, soluble tannin content of the aqueous tannin solutions. STC denotes the STC addition level.
